# Supplementary material for: Control of Slc7a5 sensitivity by the voltage-sensing domain of Kv1 channels
Source: eLife. 2020 Nov 9;9:e54916. doi: 10.7554/eLife.54916 (PMC7690953; doi:10.7554/eLife.54916)
Supplement: Supplementary file 1. [file elife-54916-supp1.docx]

| Primer list: | | |
| --- | --- | --- |
| *Kv1.1/Kv1.2 Chimeras:* | | |
| Kv1.1-Forward | GGGCTCGAGATGACGGTGATGTCTGGGGAG |  |
| 1.1/1.2-S1 | GAAACTCATTTTCAGGCAGGGGGCGCTCCTCCTCCTTGATGAAGC |  |
| 1.1/1.2-S2 | CTCTACGATGAAGAAAGGGTCTGTGAAGATGTTGGAATTGTAG |  |
| 1.1/1.2-S3 | GCCACAATGTCAATGATGTTCATGATGTTTTTGAAGAAGTCCGTCTTGC |  |
| 1.1/1.2-Pore | GGGGAACTGGGAATCTCGCTCATCCGCCTCGGCAAAGTACACTGCACTAG |  |
| Kv1.2-Reverse | GGGGAAGCTTTTCAGACATCAGTTAACATTTTGG |  |
| *Kv1.2(Kv1.1VSD) Chimera:* | | |
| Kv1.2-Forward | GGGCTCGAGATGACAGTGGCTACCGGAG |  |
| Kv1.2 N-term Reverse | CCTTCTCGGGCAGAGGACGTTCTTCTTCCTTGATATAG |  |
| *Kv1.1(Kv1.2VSD) Chimera:* | | |
| Kv1.1 Reverse | CCCGGATCCTTAAACATCGGTCAGTAGCTTG |  |
| Kv1.1 Pore Forward | AGAGAGCTAGGGCTGCTCATC |  |
| *Kv1.2(Kv1.1-S3/S4) Chimera:* | | |
| Kv1.2(1.1-S3/S4)-forward | ATAGCTGAGCAGGAAGGAAACCAG |  |
| Kv1.2(1.1-S3/S4)-reverse | CCTTCCTGCTCAGCTATCTCTGTCCCCAGGGTG |  |
| *Kv1.2/Kv1.5 Chimeras:* | |  |
| Kv1.5-Reverse | GGGTTCGAATCACAAATCTGTTTCCCG |  |
| S1 chimera | GAGAGCTCTGGGTCCGCG (breakpoint Kv1.2 154/Kv1.5 241) |  |
| S3 chimera | ATGAACATCATCGATGTG (breakpoint Kv1.2 255/Kv1.5 347) |  |
| Pore chimera | ATCTTCTTCCTCTTCATC (breakpoint Kv1.2 332/Kv1.5 438) |  |
| C-terminal chimera | CACCGGGAAACCGATCAC (breakpoint Kv1.2 417/Kv1.5 523) |  |
| 1.5S1F | GAGAGCTCTGGGTCCGCG |  |
| 1.5S1R | GGTCTCCAAGCAGAAGGTG |  |
| 1.5S1S2F | GAGTTCAGGGATGAACGTG |  |
| 1.5S1S2R | CCTGGGCAGGAGCGGTGCCAC |  |
| 1.5S2F | ACCCTGGCCGACCCCTTCTTC |  |
| 1.5S2R | GCAGGCGAAGAAGCGCAC |  |
| 1.5S2S3F | CCCAGCAAGGCAGGGTTCTCC |  |
| 1.5S2S3R | GAAGTAGGGGAAGATGGCCAC |  |
| *Point Mutants:* | |  |
| Kv1.1[Y379T] | CGGTCACATGACCCCTGTGACAATTG |  |
| Kv1.1[V168I] | GGGCCCGCCAGGATCATCGCCATCGTC |  |
| Kv1.1[V168A] | GGGCCCGCCAGGGCCATCGCCATCGTC |  |
| Kv1.2[P161S] | GAGCTCAGGGTCTGCCAGGATCATAG |  |
| Kv1.2[I164A] | CCGGCCAGGGCCATAGCCATTG |  |
| Kv1.2[M171L] | GTATCTGTGTTGGTCATTCTG |  |
| Kv1.2[V178I] | GATCTCGATCATCAGCTTCTGTC |  |
| Kv1.2[S179T] | CTCGATCGTCACCTTCTGTCTG |  |
| Kv1.2[I257F] | CATCATGAACTTCATTGACATTGTGGC |  |
| *ShRNAs:* | |  |
| ShR1 | GCAATATCACGCTGCTCAA |  |
| ShR2 | GCAGAAGTTGTCCTTTGAA |  |
| ShR3 | GGAACATTGTGTTGGCTTTG |  |
| ShR4 | GCATTGGCTTCGCCATCAT |  |
| Negative control | GCAGTTATCTGGAAGATCAGG |  |
